# International Health and the Limits of its Global Influence: Bhutan and the Worldwide Smallpox Eradication Programme

**DOI:** 10.1017/mdh.2013.63

**Published:** 2013-10

**Authors:** Sanjoy Bhattacharya

**Affiliations:** Department of History and Centre for Global Health Histories, University of York, Heslington Campus, York YO10 5DD, UK

**Keywords:** Bhutan, World Health Organization, Smallpox eradication, Vaccination, International health, India

## Abstract

Histories of the global smallpox eradication programme have tended to concentrate on the larger national formations in Africa and Asia. This focus is generally justified by chroniclers by the fact that these locations contributed a major share of the world’s annual tally of variola, which meant that international agencies paid a lot of attention to working with officials in national and local government on anti-smallpox campaigns in these territories. Such historiographical trends have led to the marginalisation of the histories of smallpox eradication programmes in smaller nations, which are presented either in heroic, institutional tropes as peripheral or as being largely shorn of sustained campaigns against the disease. Using a case study of Bhutan, a small Himalayan kingdom sandwiched between India and China, an effort is made to reclaim the historical experiences in small national entities in the worldwide smallpox eradication programme. Bhutan’s experience in the 1960s and 1970s allows much more in addition. It provides us with a better understanding of the limited powers of international agencies in areas considered politically sensitive by the governments of powerful nations such as India. The resulting methodological suggestions are of wider historical and historiographical relevance.

## Introduction

The worldwide programme to eradicate smallpox started gathering momentum in the latter half of the 1960s, after the creation of an energetic coordinating body at the World Health Organization headquarters in Geneva (WHO HQ). This office was able to mobilise unprecedented levels of political and financial support internationally. Whilst the successes in Western Africa had raised the profile of the fight against the disease in administrative circles within the United States of America, it was obvious to almost everyone supportive of smallpox eradication that a genuinely global effort would need to be rooted in the South Asian subcontinent; here countries such as India, Pakistan and Bangladesh regularly contributed the bulk of the world’s cases of variola.^1^ The West African campaign, which was coordinated by the United States Centers of Disease Control (CDC) and supported by the United States Agency for International Development (USAID), coincided with efforts to crank up the effectiveness of existing smallpox eradication programmes in South Asia (activities that were spurred on by funds provided by the WHO and the governments of India and Pakistan). This included some successful pilot projects in India with support from its federal and state governments; it is, therefore, difficult to make definitive statements about whether one regional campaign helped develop another, even if some institutional histories and memoirs seem extraordinarily confident in their claims in this regard.^2^

With Donald A. Henderson as Director, the WHO HQ-based coordinating unit entered into detailed negotiations with national governments across the world. The support mobilised ebbed and flowed over time, and this experience presented WHO officials with a steep learning curve, and forced them to face up to several harsh lessons about the complexity of local administration and politics. These experiences, as well as the difficulties created by largely autonomous WHO Regional Offices that were divided about the wisdom of providing unquestioning backing to the goal of smallpox eradication, helped Henderson’s unit recognise the need to remain adaptable towards the crystallisation of multifaceted national programmes.^3^ This enabled his associates and him to advocate a less top-down style of management, which was welcomed at least by some international workers deployed by the WHO worldwide. These were WHO nominees who had been consistently open to exchanging ideas with medical, paramedical and health officials responsible for running local administrative structures; people who generally refused to make rash presumptions about the abilities and attitudes of governmental staff, chose their local allies carefully and were adept at fostering community stake-holding. New and more reliable information flows about the actual levels of smallpox incidence were a result, which helped the production of a more accurate picture of the challenges facing the eradication programme in myriad locales. Where implemented, these policies allowed for the more efficient distribution of personnel, vaccines and funds; despite the ups and downs of individual projects, these trends contributed significantly to the falls in smallpox incidence across South Asia that were witnessed between 1970 and 1975.^4^

However, it would be incredibly simplistic to assume that these changes were embraced both universally and uniformly, and that all international workers behaved in the same way; like the efforts of their governmental counterparts, the work carried out by overseas personnel was marked by varying levels of commitment, openness and efficiency. It is also important to remember that the experiences within smaller nations in the South Asian subcontinent such as Bhutan, Nepal and Sri Lanka were rather different from those witnessed in India and Bangladesh, which were major reservoirs of the variola virus. Specificities in relation to geographical factors, national politics, international pressures, social profiles, and infrastructural and economic conditions were important determinants of how smallpox eradication programmes were developed and run. Yet, there is surprisingly little recognition of the distinctiveness of national and local campaigns across the South Asian subcontinent in the generalising narratives available to us.^5^ In relation to Bhutan, the focus of this article, it is worth noting here that the official WHO histories have struggled to provide more than a few paragraphs or pages on its experiences, and that the accounts are not particularly fulsome. For instance, the flagship history of the Indian case study prepared by the WHO Regional Office for South East Asia (WHO SEARO), which was published in 1979, manages to describe the entire history of the kingdom’s programme in the following paragraph:

From 1954 to 1965 no smallpox case was reported in Bhutan. In 1966 forty cases resulting in 20 deaths were detected. This outbreak resulted from importation of infection among newly recruited labourers coming from India. Apart from one importation in 1974, no other smallpox cases have been reported during the period 1967–75. In view of the recent endemicity of smallpox in neighbouring countries and the relatively free movement of the population between India and Bhutan, an intensified smallpox surveillance programme was organized in the second half of 1976. In autumn 1976, surveillance activities were mainly concentrated in the upper and middle zones, including seven urban areas where a house-to-house search for cases was organised together with a facial pockmark and vaccination scar survey. Weekly markets were visited periodically and outbreaks of fever and rash were investigated. At the end of 1976 and the beginning of 1977, surveillance activities were concentrated in the lower zone, bordering India. A thorough house-to-house search, covering all villages and municipalities, was organized and a facial pock mark and vaccination scar survey was carried out. Fever and rash rumours were collected and subsequently verified by experienced field workers. A WHO International Commission confirmed on 22 April 1977 that smallpox had been eradicated in Bhutan.^6^

There is nothing unusual about the brevity and blandness of this analysis. The tone was almost replicated in the multi-authored, widely celebrated official history titled *Smallpox and its Eradication* published by the WHO HQ in 1988. Numbering all of 1460 pages, it devoted about four paragraphs and a few stray sentences to Bhutan, largely placed in a concise section that also discussed the situation prevalent within the kingdom of Nepal and the Indian protectorate of Sikkim (the latter became a formal part of the Republic of India in 1975). The potted history of the Bhutanese programme provided by the following paragraph gives us an effective appreciation of the limited scope of this retrospective official analysis:

Until 1961, no health department had been established in the country. In 1964, the government created 19 posts for vaccinators, and increased the number to 25 in 1966, when a mass vaccination campaign was begun following an outbreak of 74 cases of smallpox in 1965–1966 in the capital city of Thimbu [*sic*]. The outbreak had begun among Indian and Nepalese workers employed in a road-building project and then spread to the local population. The number of vaccinations reported to have been performed between 1967 and 1975, however, was small in relation to the population of 987,000 (1967 estimate). After the 1965–1966 outbreak, only 4 further outbreaks were reported. In 1967, 2 outbreaks originating in Assam caused 14 cases. The third outbreak, of 6 cases, occurred in April 1973 in a village near the south-western border with India, the initial case having been infected on a tea estate in West Bengal. The fourth outbreak, near the same border area, occurred in February 1974 and consisted of 3 cases, of which the first had been infected in Assam. Surveys conducted in 1976 to detect individuals with facial pockmarks, as well as interviews with village officials, indicate that other, unreported outbreaks had occurred although none had produced more than a few cases. This was attributed in part to the fact that the villages were scattered and isolated, and in part to the sensible traditional practice of isolating the patient and his family at the onset of illness in a place some distance away from the village. In these circumstances, the spread of smallpox was difficult.^7^

These official narratives provide the strongest possible justification for the preparation of an alternative, more complex analysis of Bhutan’s national smallpox eradication campaigns and the many ways in which they were linked to the worldwide programme targeting variola. The most effective means of doing this is by using a range of unpublished documentation that has been stored away in the recesses of the WHO archives in Geneva, a large proportion of which has not been assessed critically before; these papers are important because they allow us to focus on debates and discussions that were largely carried on away from the public gaze. For our purposes here, it is useful to analyse an expansive set of conversations involving a variety of actors: the smallpox eradication unit within the WHO HQ in Geneva, a group of well-connected medical volunteers based in the USA, the royal court and government of Bhutan in Thimpu, the WHO SEARO offices in New Delhi and, not least, the Indian federal authorities and their representatives in the Himalayan kingdom.

## Opening Up

Landlocked between India and China, the Himalayan kingdom of Bhutan has always had enormous strategic importance for these politically and militarily ambitious neighbours. However, India benefited from treaties negotiated during British colonial rule, which allowed it to retain prolonged control over Bhutanese foreign and defence affairs. This arrangement was formalized in August 1949, a mere two years after Indian independence, through an Indo-Bhutanese Accord; this agreement allowed for the return of territories annexed by the British empire and the provision of annual subsidies by the Indian authorities to the kingdom (the terms of this Accord were only renegotiated as recently as February 2007, when Bhutan was allowed an increased say in its own external affairs and defence procurement policies). The 1949 treaty was intended to officially enshrine an arrangement whereby India did not interfere in Bhutan’s internal affairs, in return for which New Delhi was given overall control of the kingdom’s foreign relations (this included an agreement that nations represented on the UN security council would not be allowed to open diplomatic missions in Thimpu). In practice, however, both parties struggled to honour this delineation of responsibilities and the resultant tensions were visible from as early as 1952, the year a reformist monarch – King Jigme Dorji Wangchuk – ascended to the Bhutanese throne. Some of these clashes were stoked by acts attributable to influential individuals such as Jawaharlal Nehru, India’s first prime minister. The Bhutan State Congress (BSC), a new political party, was formed in India in 1952 with Nehru’s patronage; however, it was banned soon afterwards by the kingdom’s government, as it was accused of being dominated by a largely Nepalese membership driven by secessionist, anti-monarchical ideologies.^8^

Bhutan’s monarch created a partially elected National Assembly in its stead in 1953, but this did not rein in the country’s political troubles. The proscribed BSC continued to be a political force, especially amongst the Nepali-speaking sections of society fighting for access to full citizenship; it remained powerful enough in 1954 to successfully call for a nationwide civil disobedience movement, which caused an official crackdown that led to the death of about 25 protestors. The Indian government insisted on intervening to resolve the resultant crisis, brokering an uneasy deal whereby the kingdom’s government promised to pass a new Citizenship Law in 1958 that provided Bhutanese citizenship to at least some residents of Nepalese origin.^9^ Ethnic relations continued to be frail in the kingdom despite these legal advances and this was exposed by political developments that affected the region in 1959, following the entry of Chinese troops into Tibet (this correlated with an increase in covert activities by American and Indian intelligence agencies in Himalayan South Asia).^10^ Although several thousand Tibetan refugees were given asylum in Bhutan that year at India’s insistence, this influx was considered to be socially and politically destabilising. The result was a new law passed by Bhutan’s National Assembly, banning further immigration from Tibet into the country.^11^ The Sino-Indian conflict of 1962 affected the kingdom deeply in a situation where the retreating Indian army detachments used its territories as one of its escape routes. China’s resounding victory in the war caused a stiffening of Indian and Bhutanese attitudes towards their northern neighbour, leading to the militarisation of Bhutan’s northern borders adjacent to Tibet; this shared fear of further invasions also contributed to Bhutan joining the Colombo Plan treaty in 1962, as this assertion of national sovereignty was seen as providing at least some level of protection from a future Chinese foray into the kingdom.^12^

It is against this complex and unstable political and social backdrop that negotiations were started between the Bhutanese royal court and Dr Pierce Gardner, who was an Assistant Professor of Medicine at the University of Florida, USA, in 1969. On the table was a scheme that would help develop the nation’s state-sponsored health services. These engagements were very much in keeping with the kingdom’s efforts at the time to open up to the world, albeit under the close scrutiny of the Indian government. Bhutan had signed up to the Colombo Plan treaty beforehand, but much of the aid provided under this scheme for the building of health infrastructure such as hospitals was provided by India. The royal government’s negotiations with Gardner were novel precisely because they dealt with a set of people not formally associated to a Commonwealth nation. Many challenges beckoned for both parties, not least as the plans under deliberation could be latched on to existing political and economic agreements; it was clear that such an unprecedented intervention into Bhutan’s health affairs would need to be funded by external sources. Moreover, very little independently verified information about healthcare structures and disease profiles in Bhutan during the first six decades of the twentieth century was available at the time; this situation was exacerbated by the fact that a major flood in 1967 that caused the wide-ranging destruction of relevant government records. The sprinkling of extant publications on the theme of medicine and health in Bhutan tended to rely on the use of piecemeal information provided by the Bhutanese authorities as a result. We can see such interpretative tendencies in an article in the *Lancet*, which was published in 1965; authored by a couple of senior British medical practitioners, who were possibly given some independent access to Thimpu because the United Kingdom was an important signatory of the Colombo Plan treaty, the article was largely uncritical about figures provided by Bhutan’s health ministry.^13^

This is what makes the records resulting from Dr Pierce Gardner’s engagements with Bhutan’s royal court both fascinating and important. As Gardner entered into detailed negotiations with the Bhutanese authorities, about a scheme titled ‘Partnership for Medical Progress’, he was presented with an opportunity to access and accumulate significant bodies of data about healthcare trends in the Himalayan kingdom. Fortunately for us, Gardner accepted that opening; even more propitiously, perhaps, he chose to share these data sets with Donald Henderson’s office in Geneva, which filed the papers away carefully (as it transpires, for long-term use by researchers who choose to study the WHO’s voluminous smallpox eradication archives). Gardner’s access to the royal family, which seems to have been sparked by the fact that he had worked in CDC-led projects outside the USA and was seen to have connections with managers of international programmes, ensured that information about health trends in Bhutan was carefully collected for him by regal decree from a variety of localities; this material was voluminous and rich in details about the district-wise breakdown of disease affecting the kingdom’s population. It was not, by any measure, an exercise intended to present an idealised image of the healthiness of the Bhutanese population. Even then, Gardner did not accept these data sets blindly. He instead chose to cross-reference this information with figures available to him in reports and papers about Colombo Plan-funded projects in the kingdom, and then came up with blunt assessments about the gaps in state-sponsored healthcare delivery. He noted, for instance, that:

According to the latest figures, Dr Tobegeyal [*sic*] has available only a handful of trained physicians plus 25 compounders (workers with two years of medical training) to assist him in providing medical care for the entire nation – 800,000 people scattered over an area of 18,000 square miles. Even so, together they have been able to staff the country’s four hospitals, with a total bed capacity of 120, and the dispensaries located in each of the 23 dzongs, or main districts of Bhutan. While significant progress has been made in improving the health care available in the country, the small number of medical personnel and facilities still restricts medical services in Bhutan.^14^

Gardner also mentioned India’s great influence on Bhutan’s public health agendas, noting that Dr P.D. Gogoi, an Indian physician, headed the relevant federal department. He made reference to a number of active health projects, but specially mentioned the significance accorded to malaria eradication work that was financed by India and focused on the Phuntsholling area in the southern part of the country. In relation to smallpox immunisation programmes, Gardner wrote about 20 vaccinators who were employed by the government after a major variola outbreak in 1966 (their work was mainly targeted at the areas to the west of the Black Mountains).^15^ He flagged up data that showed that diphtheria, pertussis and BCG vaccines were available in dispensaries, but there was little evidence to show that routine preventive work was carried out in relation to these diseases. Sexually transmitted diseases, as well as health problems created by worms and diarrhoea, were widely prevalent in the kingdom.^16^ Gardner also mentioned campaigns against goitre, which were discontinued for a couple of years due to unspecified socio-political problems.^17^ Data available to him revealed that the anti-goitre work was based on the general introduction of iodized salt.^18^

These details provided all the parties involved – Donald Henderson and his colleagues in WHO HQ included – with insights into the workings of a country about which very little was known within the wider community of international health. The materials were wide-ranging and detailed enough for Gardner to tabulate figures, formulate generalisations, set targets and forward proposals for collaboration to the WHO HQ. The scheme he suggested to Henderson was a broad-ranging one, as we can see from an extract drawn from one of his draft plans:

In paving the way for improved, long-term health standards…[mobile health] teams have been more effective when acting in partnership with indigenous personnel in carrying out a specific health project…. It is clear…awareness of and desire for public health programs exist in Bhutan. But the forceful prosecution of the plans…require[s] a more effective delivery of medical care than that currently available in the Kingdom. We believe that even a small number of medical personnel, if organized into mobile health teams, can help Bhutan achieve a more comprehensive program of disease prevention and public health education. Hence, we propose to make available to the Kingdom of Bhutan physicians and paramedical personnel for the purpose of demonstrating the practicability and desirability of setting up such mobile health teams.^19^

The scheme was to be built around the establishment of three teams, each composed of members of Gardner’s American colleagues working alongside Bhutanese personnel. Smallpox vaccination was intended to be the primary focus of the programme, as Gardner and his colleagues hoped that these were likely to develop into a series of locally supported projects over the long term. The entire activity was planned for March, April and May 1970; this was to include a two- to three-week training stint in the cities of Thimpu or Paro aimed at creating synergies between overseas and local team members. The expectation was that the success of this pilot would cause immunisation services to be included in wider, more permanent mobile disease prevention campaigns.^20^ Gardner hoped that financial support for the planned scheme would be provided by non-government sources such as private foundations and non-profit organisations; he did not intend to ask the Bhutanese government for financial contributions and expected its role to be limited to employing the local personnel who would join the mobile health teams and provide donations of equipment (in the shape of a vehicle and some camping equipment for each team).^21^ The costs calculated for the partnership, outlined in minute detail by Gardner in a document titled ‘Supplement Report – Bhutan’, were not enormous by the standards of the time for international collaborative efforts launched in developing countries: a most carefully calculated sum of US$26,814.80. Considered valuable by King Jigme Dorji Wangchuk and Prince Namgyal Wangchuk, the launch of the scheme was formally invited from by the Bhutanese government in June 1969.^22^

A letter from Bhutan’s Ministry of Trade, Commerce and Industries to Gardner that month informed him that the expectation was that the proposed project would continue for at least twelve weeks, that the main geographical focus of the teams would be central Bhutan, that the costs of travel to the Kingdom and local maintenances expenses would have to be met by the team itself, and that the arrangements for vaccine and medical supplies would also need to be their responsibility. In return, the Bhutanese authorities offered to supply vehicles and fuel for travel, and enlist compounders, medical and paramedical personnel to become members of the proposed mobile teams.^23^ It was now time for Gardner to turn to Donald Henderson, with whom he had a discussion previously about his plans at an Epidemic Intelligence Services meeting at the CDC offices in Atlanta, for assistance. The correspondence between the two men tells us a lot about the complexities bedevilling the smallpox eradication programme and the wider international health landscape; features that we would never be aware of without careful primary research in the available archives.

## A Stunted Foray

Bhutan started to upgrade its smallpox control facilities around the mid-1960s in response to a series of outbreaks linked to Indian workers employed in road building projects across the kingdom.^24^ Very little seems to have been known about these immunisation efforts within WHO HQ and WHO SEARO for the bulk of the decade, due mainly to a culture of secrecy relating to the administration of Bhutan that was jealously guarded by the Indian government; a state of affairs that United Nations (UN) agencies, including the WHO, could do little to change (Bhutan only joined the UN in 1971). However, Pierce Gardner’s correspondence began to provide Donald Henderson and his team within the WHO HQ with unprecedented detail about variola incidence in the kingdom. In a letter to Henderson, sent in April 1969, Gardner repeated that he had been invited by the Bhutanese royal family to start a medical programme that was tentatively slated for the spring of 1970. The missive made a case for an assessment of the effectiveness of mobile health teams, mainly as Gardner noted that data available to him showed that Bhutan had limited numbers of medical personnel and that it was important to provide immunisation facilities outside urban areas. As an avid advocate of preventive health, he wondered if an immunisation campaign against smallpox might be made the central part of the planned project. At the same time, Gardner also highlighted his support for a suggestion made by Henderson during their talks in Atlanta that the smallpox and BCG vaccines be administered simultaneously in Bhutan. He added that:

I greatly appreciate your offer of support in the form of vaccine and in terms of possible future consultation. We are currently in the fund raising stage, approaching various foundations and drug companies. It would be helpful to have a letter from WHO stating that this project is in keeping with the goals of the WHO eradication program, that it is a scientifically sound program and that you would encourage its support. I also plan to contact UNICEF concerning the possible use of BCG in the program.

Gardner signed off his letter with a hand-written postscript requesting an outline of the pox survey techniques that Henderson had mentioned in Atlanta as being useful for baseline data gathering in the smallpox eradication programme.^25^

Gardner persisted despite not receiving an immediate reply and shot off another missive to Henderson the following month. This letter underlined the point that he was poised to make a preliminary visit to Bhutan in the first half of June and that he would be presenting his programme proposals to the kingdom’s government. He requested the name of the WHO SEARO representative who could be approached for assistance and asked, once again, for ‘a description of the scar survey technique that was worked out in West Africa’.^26^ This persistence paid off. In a personal letter, warm in tone, Henderson apologised for the delay in replying, saying that he had been travelling constantly. Henderson provided Gardner with Dr Jacobus Keja’s name as the person to contact in relation to matters regarding smallpox eradication within WHO SEARO, which was responsible for negotiations with the Bhutan government on health-related issues, and enclosed three copies of the forms he used for scar surveys. Significantly, Henderson also advised him about the importance of embracing techniques that had been developed for use inside the region, declaring that:

The methodology employed has varied rather considerably from country to country and we do not, in fact, have a written protocol with regard to methodology as it is presently being applied. I would urge that you talk further with Dr Keja about this for the approach which he is using in South-East Asia appears to be both simple and effective although not qualifying as a valid statistical sample which introduces all sorts of complexities in the developing countries.^27^

This was to be one of many reminders, from Henderson to outside observers, that epidemiological models that had been advertised as being successful by smallpox workers involved in West Africa were not being blindly utilised in disease hot spots such as South Asia. Indeed, in the late 1960s, core strategies for developing a wholesome understanding of smallpox incidence in Asia were based on an adaptable set of scar survey policies that were variously implemented in different localities. Henderson also underlined the WHO’s inability to disregard regional, national and local political frameworks during the design and implementation of policy, even as he sought to assure Gardner that he would do his best to mobilise the necessary smallpox vaccine and bifurcated needles. Henderson was, in fact, quite optimistic in June 1969 when he declared that:

In the smallpox eradication programme, we are most happy to have the participation of all who might contribute to it. As you will undoubtedly understand, our relationships must ultimately be dealt with through the individual governments. In order for us to provide vaccine and bifurcated needles, I would need to have a request from the Government of Bhutan. I would gather from your letter that this would not be a difficult problem to arrange in consultation with them. Following receipt of a request, arrangements can be made to despatch both vaccine and needles very rapidly, although what sort of transport is available into Bhutan at this time, I simply do not know. Clearly, we could have the necessary supplies in Delhi within a period of a week, as vaccine is stored in Geneva along with the needles.^28^

Whilst acknowledging the importance of considering the political expectations of national governments, this correspondence revealed that Henderson had not been fully aware of the diplomatic protocols in relation to dealings with Bhutan and India. He was soon disabused of the view that it was appropriate to deal with Thimpu without WHO Regional Office participation. The evolution of Henderson’s understanding about the care needed whilst dealing with Bhutan’s authorities is demonstrated most effectively through his correspondence after Gardner reported an outbreak of about 2000 cases of smallpox in 1966; a detailed breakdown of cases had been provided to him by the Bhutanese health ministry at the request of the royal court, and he had forwarded this to the WHO HQ. Henderson was horrified by these figures as these details had found no place in the epidemiological information provided by WHO SEARO to his unit in Geneva (this was a major outbreak by contemporary epidemiological measurements). Once again, apparently unaware of the political sensitivities in the region, he asked Gardner in July 1969 if it would be possible for him to request the Bhutanese authorities to provide details of smallpox outbreaks to the WHO HQ on a regular basis, adding that this ‘would be most helpful’ to the progress of the worldwide eradication programme. However, Henderson was, by this time, much more measured in his advice to Gardner about dealings with WHO SEARO and the best means of arranging a request of aid for the kingdom, as the following extract from a letter shows:

I am pleased to hear that your programme has been accepted by the Government of Bhutan. I have since received a note from our office in Delhi indicating that the external affairs of Bhutan are handled by the Government of India. Considering this, I think it most important that the Government initiate a request for vaccine and needles at the earliest possible time. Indian bureaucracy is not noted for its speed. A decision with respect to WHO paying a consultant to Bhutan would have to be made by the Regional Office. I doubt that they would be very receptive to proposal that one of the members of your team be supported as a WHO consultant as a sort of fait accompli. I would hope that you would be able to find support elsewhere. As a last resort and probably as a very long shot, we might give it a try but I would not be terribly optimistic.^29^

Henderson continued to be supportive of Gardner’s planned enterprise in Bhutan at this point of time, which was clearly demonstrated by much more than the correspondence between the two men. A personal memorandum from Henderson to the Regional Director at WHO SEARO underscored his office’s enthusiasm for Gardner’s proposals, adding, unambiguously, that it was ‘appropriate and wise’ to help the project with vaccine supplied by the WHO. The tone of the rest of the message was more awkward. He forwarded Gardner’s findings that approximately 2000 people in Bhutan had been affected by an outbreak of smallpox in 1966, also noting that Gardner was helping his unit in Geneva develop a detailed assessment of Bhutanese immunisation facilities and the epidemiological situation. He concluded the message to the Regional Director by suggesting that ‘I would hope that we would be in a position to contact Bhutan to request that they submit regularly to WHO report of smallpox so that we will have some better appraisal of what is going on’.^30^

The correspondence trail between Gardner and Henderson went cold for a while, but picked up again in February 1970. Having received a letter from Gardner, who was now serving at the Children’s Hospital Medical Center in Massachusetts, dated the 12th of that month, Henderson responded in detail. He was much less enthusiastic and hopeful about the plans for Bhutan by now, having been briefed about the political and institutional challenges in relation to the region; indeed, he was very frank about the local obstacles when he argued that:

Bhutan depends on India for its foreign relations and thus any sort of assistance on the part of WHO would require some sort of formal request, I presume, to the Government of India. I rather doubt that WHO would look very favourably on sponsorship of a very short term mission such as this, as experience would suggest that the lasting impact of such a programme would not be great. If under WHO auspices, I am sure that the Organisation would demand a major voice in the planning and policies. There is another and very major problem and that relates to obtaining acceptance of any such proposal from the Government of India. Our experience has been that deliberations are rather slow and to obtain agreement on comparatively simple matters such as approval of a resident of India to attend a WHO conference at the Organisation’s expenses often requires months…. Personally, I believe it would be a significant contribution to the progress of smallpox eradication were you to conduct such a vaccination scheme in co-operation with the Government [of Bhutan]. The realities of international relationships are such, however, that any such project such as this is a highly involved problem and thus I would encourage you to take the simplest possible route realizing that even this will not be simple.^31^

A private memorandum from Henderson to the Regional Director at WHO SEARO reveals how he was gradually distancing himself from Gardner’s plans for Bhutan by this juncture, describing them as being ‘rather ill-defined’; this may well have been engineered by a general acceptance inside the WHO that is was not worth annoying the Indian authorities so soon after they had to be convinced to remain committed to the worldwide smallpox eradication programme.^32^ What was remarkable, however, was that Henderson still valued the information contained inside the documents put together by Gardner for the WHO HQ, presented them to WHO SEARO as being valid and then sought to use these as a basis for suggesting policy reform inside Bhutan. Having forwarded these papers to WHO SEARO, he underscored the importance of the detailed epidemiological information that suggested that there had been about 1900 cases of smallpox in the kingdom during the course of 1966, reiterated the fact that the WHO SEARO had not recorded these smallpox cases officially, and enquired whether if it was possible to use this data to develop more effective disease surveillance and vaccine distribution programmes for the kingdom.^33^

**Table 1:** Smallpox epidemic in Bhutan in 1966: breakdown of cases. Source: Pierce Gardner, Table 1 in ‘Supplement Report – BHUTAN’, *c*.1969, Folder 55, Box 184, WHOHA.

	January	February	March	April	May	June	July	August	September	October	November	December	Total
Thimpu	–	–	–	–	–	–	–	–	–	615	–	–	615
Paro	–	–	–	–	–	–	–	–	–	–	–	–	0
Phuntsholling	–	–	–	–	2	–	–	–	–	–	–	–	2
Samchi	4	7	–	4	19	–	–	2	–	–	–	–	36
Sarbhong	1	24	8	13	7	1	–	–	–	800	–	–	853
Byagar	–	–	–	–	–	–	–	–	–	–	–	–	0
Tashigang	–	–	4	327	5	–	–	58	–	–	–	–	394
Total	5	31	12	344	33	1	–	60	–	1415	0	0	1900

There is no record in the otherwise bountiful WHO archives in Geneva to suggest that the Regional Director at WHO SEARO bothered to respond immediately to Henderson’s entreaties in relation to Bhutan, let alone allow his recommendations to guide the reformulation of his office’s policy. Instead, the Regional Director’s office, which was angered by this criticism, chose to draw a veil of silence on the matter over the course of several months; their annoyance was exacerbated by further doses of prodding from Henderson and his associates based inside a dedicated smallpox eradication unit that was set up within the WHO SEARO. The Regional Director’s office stalled a discussion for almost two years and the reply, when it arrived, advocated a completely different set of smallpox figures for general consumption. These were presented by Dr P.W. Samdup, Bhutan’s Superintendent of Health Services, and backed by the Indian authorities, in a major meeting sponsored by WHO SEARO in October–November 1972.^34^ This interpretation represented a most dramatic rebuttal of the figures of smallpox incidence unearthed by Gardner with the assistance of the Bhutanese royal family and highlighted by Henderson inside the WHO, when it declared that:

As our records show, we had no outbreak of smallpox from 1954 to 1966. Before 1954, we had no records. In early 1966, four imported newly recruited labourers, coming via India, arrived in a border town with fever. The next day they proceeded to their camps, which are about 200km from the border. They reached their camp in two days. By then, they had more fever and some rash. On the fourth day after their arrival in Bhutan, they were brought to the hospital in Thimpu, where it was clinically confirmed that they had smallpox. With full cooperation of the public and the Government, we could control the outbreak in four months time. There were a total [of] forty admissions in the hospital, of which twenty survived. Since then Bhutan has been free of smallpox.^35^

Gardner’s proposed scheme was shelved for good around the same time as this announcement. Apart from the inability of WHO HQ and WHO SEARO to provide the necessary infrastructural assistance, there is no evidence to show that the Indian government’s help was ever requested on his behalf by WHO sources based in Geneva or New Delhi. Nor was support from the Indian authorities directly forthcoming to Gardner and his colleagues. King Jigme Dorji Wangchuk’s prolonged illness and death whilst receiving treatment in Kenya in 1972 proved to be the final, most damaging blow to Gardner’s plans.^36^ Wider political developments in this period would, of course, have made it incredibly difficult for an American citizen, unaffiliated to a neutral UN agency and intending to volunteer in Bhutan to salvage the situation. The tensions between India and the United States spiked in 1971 and several issues were responsible, although two were most prominent: a civil war in East Pakistan that would lead to a fully blown conflict between India and Pakistan, and the creation of the sovereign nation of Bangladesh. The political disagreements occurred at many different levels. On the one hand, India was unhappy with the US’s unwillingness to denounce the Pakistani army’s atrocities against the religious minorities and Bengali nationalists in the eastern wing of the country, the military aid provided by her to Pakistan and the Nixon administration’s ambivalence towards China.^37^ On the other hand, there was anger inside the US government about India’s unilateral deployment of Tibetan armed units in this conflict. These militias had been trained jointly by America’s and India’s intelligence agencies for undercover missions inside Tibet; the withdrawal of US military and financial assistance for this covert programme, during the course of 1971 and 1972, signified a nadir in relations between the two countries.^38^ The effects of these trends on the regional smallpox eradication programmes were long lasting. A host of bilateral agreements were put under pressure or discontinued, and international health projects were not unaffected. There were, for instance, concerns inside WHO frameworks as late as 1973 about the effects of the leaching away of American monies that had been used to pay the salaries of people working within India’s smallpox eradication programme, as the fortunes of its constituent campaigns were of wider regional relevance. One internal WHO communication suggested the following approach to getting India to accept US aid, which could then be used to strengthen anti-smallpox campaigns:

It seems that the Americans might welcome such a suggestion [of offering rupee funds that it had accumulated from selling wheat to India as aid], and if you are able to interest them in Geneva to offer it before the Indian Government, it might very well be accepted here. I doubt that the Indian government would actually advance such a suggestion, because it really is what the PL 480 funds [money from the wheat sales] were designed for, namely to allow American ideas of ‘AID’ to alter the Indian internal domestic situation with massive amounts of rupees. But they really may accept it if it were offered as a ‘concession’ by the USA.^39^

The civil war in East Pakistan, and the conflict between India and Pakistan that followed, had other effects on smallpox eradication activities across South Asia, including the kingdom of Bhutan. The mass influx of Bengali refugees into the Indian states of Assam and West Bengal created severe humanitarian crises that necessitated wide-ranging international action and led to the dispersal of resettlement camps across India (sometimes in remote locations that were not to the liking of the refugees themselves).^40^ On several occasions, it was alleged that unvaccinated refugees had imported smallpox into the rehousing centres in West Bengal and Madhya Pradesh, even though this contradicted the claim of WHO and government authorities in East Pakistan/Bangladesh, who insisted that they had been able to stamp out the disease. The events in the Salt Lake Refugee Camp on the outskirts of Calcutta were a good case in point; supervisors there reported a series of smallpox importations in early 1971, claiming that these had acted as the basis of further outbreaks in other parts of West Bengal state, such as the 24 Parganas district.^41^ Similar reports were also received in relation to concentrations of refugee populations in Assam and Madhya Pradesh state.^42^

The WHO openly acknowledged the difficulty in monitoring the movements of Bangladeshi refugees inside India, not least as some of them chose not to stay on in the resettlement camps they had been allocated. Whilst these problems were not openly discussed between the Indian government and the WHO, both parties were realistic enough to recognise that these population flows could create serious difficulties for the efforts to contain the spread of variola. As a result, agreements were reached to create schemes that would allow an increase in the rigour of surveillance, containment and vaccination activities in the eastern and north-eastern wings of the country.^43^ However, the WHO Smallpox Eradication Units in Geneva and New Delhi soon had their ambitions clipped by a powerful and unrelenting Indian government. Whilst New Delhi proved to be unusually flexbile about special campaigns on Bangladesh’s western borders with India – a mark, perhaps, of the great confidence it had in the new, pro-India Bangladeshi regime – WHO access to the wider region was denied. WHO employees were generally not allowed to take charge of smallpox operations in districts in eastern India with international borders (the permissions given to Dr Claudio Amaral, a Brazilian public health expert working for the WHO on the Indo-Bangladeshi border, were exceptional, although this seems to have been caused by wide-ranging local political goodwill towards him rather than any masterstroke of WHO SEARO diplomacy).^44^ To add to the WHO’s frustrations, there was a complete ban on visits by workers from overseas or those in foreign employ to Manipur, Nagaland, Tripura, Mizoram, Meghalaya and the North Eastern Frontier Agency (later renamed Arunachal Pradesh). A WHO SEARO memorandum argued that this threatened the regional coordination of smallpox eradication efforts, adding that:

We have official reports…of active smallpox in these areas and unofficial reports of much more extensive smallpox outbreaks, which pose very grave international threats to neighbouring countries of Burma, Bangladesh, China and Nepal. We have already received three unofficial reports of possible cases of smallpox in Bhutan…. We are not able to place a WHO medical officer of Indian nationality in these areas and therefore we feel it most urgent that Dr Mahler specifically ask the highest Government of India authorities which nationality WHO staff will be acceptable for posting in these areas and to press for a WHO presence there in the autumn campaign….^45^

As it transpired, the efforts to lobby the Indian government on this count failed. Dr Karan Singh, the federal health minister, was opposed to an increase in the numbers of international personnel. Henderson was sent a frank assessment of the problems in this regard in 1973, which noted that:

With every passing week it is going to be more difficult to get the required number of people in place before spring…. Yesterday, meeting with Diesh, Basu and M.S. [M.I.D. Sharma], even though they allowed for 3 CDC types, it was clear that they can’t increase international staff even if they want to because they feel the new minister does not want international staff…. Therefore, there is no sense of urgency where it is needed, there is no concept of the quantity of personnel required and we have reached the end of what can be done from our level. We need Geneva level staff to push this immediately, as even agreement in January [1974] will not result in people in the field for 1 to 2 months. Since it is not possible for you to come before the end of the month, is there any possibility that Dr Mahler could spend 2 days in Delhi to sell this? This would have to be done with the Minister or even the Prime Minister….^46^

Moreover, India’s Health Minister and Ministry of External Affairs refused to entertain any WHO personnel in north-eastern India, Bhutan and the districts in Assam bordering the kingdom; instead, the WHO had to accede to the Indian government’s insistence that it accept the findings of its officials serving in these areas. India’s ascendancy over Bhutanese affairs was underlined in all negotiations carried out with WHO HQ and WHO SEARO between 1972 and 1975, and New Delhi promised to make data about variola incidence in the kingdom available to the Regional Office at regular intervals. Despite the persistence of reservations inside the WHO about the integrity of this information, its officials could do little more than to receive, tabulate and publish the data about smallpox incidence and vaccination figures that were submitted by teams of Indian and Bhutanese workers. In the process of making sense of this data, WHO personnel were generally left with little choice but to accept the accompanying assurances of everything being in order in the Himalayan kingdom. This meant relying on reports that spoke of continued mass vaccination along the borders with India, more selective vaccination elsewhere, surveillance efforts helped along by army units based in central and northern Bhutan, and the odd unverified mention of a case of smallpox. The datasets varied in scope. Some dealt with information about a specific year, whilst others provided a longer term perspective on smallpox epidemiology in Bhutan; there were some variations in the latter collections and, quite remarkably, certain documents acknowledged the existence of a major outbreak of variola in 1966.^47^ The data unearthed by Pierce Gardner in 1969 was, perhaps, not as inaccurate as it was made out to be in New Delhi during November 1972.

## The Empowered Interpretations

Material available in the WHO’s voluminous archives shows that the insights provided by the rich data collected by the Bhutanese authorities for Pierce Gardner – details that were then forwarded to WHO HQ and WHO SEARO – were removed from public gaze as preparations for the certification of smallpox eradication were started in Geneva and New Delhi. Instead, alternative, politically acceptable and, significantly, mutually sustaining narratives of what had happened in Bhutan were created by parties that were in close contact with WHO SEARO. The common thread in these interpretations of the incidence of variola in the Himalayan kingdom, and the shape and timing of the measures deployed to control the outbreaks of the virus, were a set of Indian government officials keen to manage the public messages that were going out about Bhutan; these were the very people who would go on to play a major role in the production of documentary evidence that would allow a supposedly international committee to certify smallpox eradication in Bhutan. These trends are well represented by an unpublished report titled ‘Operation Smallpox Zero Bhutan’, which was presented by the Bhutanese government in March 1977. The document seemed duty bound to report the breadth of Indian input at the very outset, declaring that:

A special surveillance programme was organised from July 1976 which included various techniques like active house to house search, facial pock mark survey, vaccination scar survey, market search, school visit, introduction of routine smallpox surveillance and enquiry of health staff about the knowledge of smallpox incidence in the country. This report covers the activities up to December 1976 [*sic*]…. I must make a mention of Dusho (Dr) P.W. Samdup, Superintendent of Health Services who was the Project Director of the ‘Operation Smallpox Zero – Bhutan’ and Dr J.L. Kole, Programme Officer, Malaria Eradication Programme, who took the leadership in organising the programme under difficult circumstances. I acknowledge the assistance from Government of India especially from Dr R.N. Basu, Assistant Director General of Health Services (Smallpox), New Delhi, Dr R.S. Sharma, Field Epidemiologist and Mr Zafar Hussain, Paramedical Assistant in [the] development of the programme and [for] keeping it going.^48^

The report went on to provide detailed descriptions of geography and terrain, climate, demographics, ethnic profiles, religion, language, religion, socio-economic data, literacy levels and educational structures, civil administration, and the migration patterns between southern Bhutan and the Indian states of Assam and West Bengal. A description of health structures followed:

A widespread belief in evil spirits as the cause of disease led to cures being sought in the performance of certain rituals by the priests (Lamas)…. It was against this background that the Health Department was established in 1961…. The whole country is divided into six zones and each zone is under the administrative control of a Zonal Medical Officer. He is assisted in the supervision of the dispensaries in his zone by a Medical Officer. Each dispensary is in charge of a trained compounder, who is responsible for all outpatient care and in many of the dispensaries, some Basic Health Units also exist numbering nine at the moment [*sic*]…. Primary and revaccination against smallpox is carried out by vaccinators throughout Bhutan using freeze-dried vaccine but using the rotary lancet and in some places with the bifurcated needle (Thimphu General Hospital is using bifurcated needle). The local name of smallpox is ‘drumne’, chickenpox is called as ‘purukachu’ and measles is ‘machum’.^49^

All this detail formed the background for a potted history of the chronology of Bhutan’s smallpox eradication programme. In this interpretation of events, the story began in June 1906, when J. Claude Smith, then British Political Officer for Bhutan, led a small team that vaccinated the populace of a village that had been stricken by the disease. The narrative then hurried onward to 1961, which was presented as a significant date since it marked the beginning of anti-smallpox work being integrated into the new structures of medicine and public health that were being created by the Bhutanese government. It was noted that nineteen vaccinators were added to the health cadres in July 1964, followed by the addition of five more such officials in December 1965. It was claimed that the next leap forward happened between 1966 and 1967, when the six Zonal Medical Officers were asked to pay attention to the creation of mass vaccination campaigns (the report noted that freeze-dried vaccine had been introduced in 1966). Further advances were claimed for 1968, when a vaccination post was created in Phuntsholling to supervise and immunise ‘imported labourers’, some of whom had been identified as a source of variola outbreaks. The WHO’s involvement was mentioned for the first time in the next entry of this historical account, which dealt with November 1972. The section described the inter-country seminar on smallpox surveillance held by WHO SEARO; this was, of course, the event at which WHO SEARO publicly embraced the epidemiological details that were provided by P.W. Samdup, then Superintendent of Bhutan’s Health Services, and ignored the data about variola incidence that had previously been forwarded by Pierce Gardner to the WHO HQ. The historical narrative went on to mention other forms of WHO support, noting that supplies of ‘smallpox surveillance materials’ were provided in March 1973. What was described as the next important step in Bhutan’s smallpox eradication campaign, dated at June 1976, made greater reference to the Indian government than the WHO:

At the request and invitation of Government of India, a medical delegation headed by Dr P.W. Samdrup [*sic*]…accompanied by Dr J.L. Kole, Programme Officer, Malaria Eradication Programme, visited New Delhi from 15 June to 19 June. The medical delegation discussed with the Director General of Health Services, Government of India and Dr R.N. Basu, Assistant Director General of Health Services (Smallpox), Government of India, the smallpox surveillance to be undertaken in Bhutan. The ‘Operation Smallpox Zero, Bhutan’ was prepared with Dr P.W. Samdrup as the Project Director [*sic*].^50^

These interventions by India did not go unrewarded. The scheme was approved by the Bhutanese government in July 1976, which led to a series of new activities. R.N. Basu, Dr T.K. Ghosh, an epidemiologist, and Mr Z.A. Arya, a paramedical assistant, travelled to Bhutan the following month, with the aim of negotiating the structure of Bhutan’s smallpox surveillance campaign. Following these deliberations, the core epidemiological team for Bhutan was identified in September 1976; this involved transferring Dr R.S. Sharma and Mr Zafar Hussain from India to the Himalayan kingdom, and they were given the task of selecting and training a handful of local officials to support the initiative. According to the historical narrative provided in ‘Operation Smallpox Zero’, this led to a series of concerted activities between September and December 1976, which were described thus:

A pre-search meeting of Zonal Medical Officers was held in Phuntsholling [in September 1976]…. The town areas of the upper and middle zones of Bhutan were covered by an active house to house search with the help of the 10 N.S.S. [National Service Scheme] personnel. A smallpox pox mark and a vaccination scar survey was combined with the active search [between September and October 1976]…. An active house to house search of the Malaria Surveillance Zone by malaria staff in Southern Bhutan was conducted. Smallpox facial pock mark and vaccination scar surveys were done among a sampled population. General training sessions on smallpox surveillance were conducted in different zones [between November and December 1976].^51^

The historical section of this report was followed by a detailed analysis of smallpox epidemiology in Bhutan. This part of the report was prefaced by an admission that the surveillance project did not ever aim to search the entire country; the search operations were mainly confined to the towns of central and southern Bhutan. The importance accorded to acquiring historical information was highlighted and this information was collected through three distinct sources: interviews carried out with health and medical staff, information collected from people in areas where searches were being carried out, and the official records of the Bhutan’s Directorate of Health Services. In practice, this meant that answers were collected from 181 medical, paramedical and public health personnel, and interviews and scar surveys were carried out on a random basis with 394 individuals from 37 villages in northern Bhutan, and during the examination of 23,316 children elsewhere in the country.^52^ The description of the available Bhutanese official records and the information provided by them is particularly fascinating, as it allows us critical insights into the ways in which specific data sets, interpretations and memories were privileged over others:

Records of a 1965–66 outbreak in Thimphu are available at the Directorate. Reports of smallpox indicating the names of areas affected with smallpox, received at the Directorate from peripheral institutions are available, and these reports mainly pertain to the period 1965–66. In each area the search teams visited a number of villages reported to hav had smallpox in the past, representing about 40% of all known smallpox villages. The Village Headman or old man, as well as the households affected with smallpox were contacted. Relevant and available epidemiological information on the smallpox outbreak was recorded. This included the year and month of the outbreak, the source of infection, the number of affected families, the age and sex of patients, the verification of surviving patients and any other information about the outbreak.^53^

Clearly, the bank of information that had been collected for Pierce Gardner by the Bhutan government in the late 1960s had been found in official repositories. But, while this data was considered reliable enough to be used to guide the fact-finding efforts of the surveillance teams, the exact figures relating to significant levels of smallpox incidence in the mid-1960s were blanked out in the report on ‘Operation Smallpox Zero’. Instead, vague references to epidemiological peaks were allowed to retain a prominent place in this interpretation of events, and a relevant and representative section is worthy of detailed quotation:

The high mortality of smallpox left a permanent memory and impression in an affected village and the people were able to give the history of even very old smallpox outbreaks including the names of patients who died and the source of infection…. There is no evidence of unbroken transmission of smallpox within Bhutan. Importations used to be contained within one or two villages and were self-contained. Earlier than the fifties, the importation was usually from Tibet due to trade links with Lhasa. Subsequently with the sealing of the Bhutan Tibet border, people from the north came for trade etc. to Assam and West Bengal in the south. During the winter months, the stay of people in weekly market areas of Assam and Bengal bordering Bhutan was for two to three months and thus importations were mainly from these market areas…. During 1965–67, with the construction of roads in East, Central and Western Bhutan, smallpox was imported through labourers from Nepal and Bihar engaged for this construction work…. 1965–66 seems to be the peak period of smallpox in Bhutan.^54^

This vagueness had a definite purpose: to propose to a specially convened international committee that was going to be tasked with certifying Bhutan free of smallpox, its primary audience, that there was nothing to worry about in relation to Bhutan despite the variability in the quality of epidemiological information. An effort was made to underscore this point with a detailed description of fever and rash surveillance efforts in the run-up to certification, which were presented as being both comprehensive and robust.^55^ Particular emphasis was placed on the effectiveness of the ‘rumour registers’ that had been introduced in September 1976:

…to record and verify all the cases of fever and rash notified to basic reporting units by active search or secondary surveillance. These registers are being maintained by Basic Reporting Unit and a monthly report in a proforma is submitted to Zonal Medical Officers with a copy to Superintendent of Health Services. The Zonal Medical Officers are required to send separate monthly reports of fever and rash cases to the Superintendent of Health Services for their respective zones prepared after receipts of reports from Basic Reporting Unit [*sic*]…. Suspected smallpox cases or chickenpox deaths are to be reported to [the] Director of Health Services and Zonal Medical Officers by wireless telegraphy, telephone and special messenger for verification and investigation.^56^

The statement on smallpox surveillance achieved its objectives, as the papers of the international smallpox assessment commission for Bhutan, which were made accessible only to select individuals at the time, show. Apart from providing information about how Indian and Bhutanese army units helped with smallpox reporting, search and vaccination work, the commission’s report claimed that the available figures allowed it to conclude that:


 The last known case of smallpox occurred in Bhutan in February 1974. Ever since, the smallpox surveillance system in Bhutan has been sufficiently sensitive to have detected smallpox transmission, should it have occurred.


 Since the requirements for smallpox eradication as defined by the WHO Expert Committee on Smallpox Eradication (1972) have been met, smallpox has been eradicated from Bhutan.^57^

However, a lot more was going on here than readily meets the eye. The composition of the certification commission for Bhutan was, by itself, highly unusual. Its membership was dictated by India, which explains why this body did not have an international member in the conventional sense, that is, an evaluator from a country that had not been deeply involved in the running of a national smallpox eradication programme, which was the general policy adopted to ensure a fair, free and critical investigation. In the evaluation of Bhutan, the position of Chair was instead given to Lieutenant General R.S. Hoon, the Director General of the Indian Armed Forces Medical Services (Hoon had also served as a member of the Indian commission, where he was given sole responsibility for reporting on the north-eastern states, where, as we saw earlier, WHO officials were denied access). The two ‘accompanying epidemiologists’ were also from India as well; strikingly, they were none other than the two officials who had been running the kingdom’s ‘Operation Smallpox Zero’ programme alongside Bhutanese officials – R.N. Basu and R.S. Sharma. These structures of evaluation were given an even more incestuous flavour by the choice of the Bhutanese personnel to work with the ‘international’ commission. Dr Samdup and Dr Yonton, the two local officials assigned to the body, were as invested as Basu and Sharma in the design and running of the smallpox surveillance teams that had been tasked with producing evidence for the commission.^58^ It was a truly extraordinary situation. A small group of men were empowered to assess the effectiveness and certify the excellence of a public health project that they themselves had created and managed. No declarations of conflict of interest were made by any of the parties, and none were demanded by the WHO HQ and WHO SEARO.

All this was a far cry from the idealistic descriptions presented in *Smallpox and its Eradication*.^59^ Whilst the book’s transparency in relation to the situation inside the territorial boundaries of the Republic of India is laudable, it avoids detailing the complexities of the certification activities in Bhutan. The interpretation provided by its authors is fascinating. The book presents a set of ideal protocols and an acknowledgement that these were abandoned in certain national contexts in response to specific circumstances. While it accepted that India was able to restrict the movement of overseas workers inside its own territories (perhaps because the existence of these strictures were so well known within the international health community), the volume failed to provide any details about why the Indian authorities were able to determine the running of the smallpox eradication programme in neighbouring Bhutan (despite the fact that the Himalayan kingdom was a sovereign nation and not officially under Indian military occupation). What the book provided, instead, is a set of non-committal and vague explanations, the rationale for which becomes clear only when one studies the way in which the composition of the commissions created to certify smallpox eradication commissions put together in different countries is described. The names of those who worked in India and Bhutan are listed together in one section of the book (without explanation), and it is easy for the unquestioning reader to assume that all those who were mentioned here were active inside both nations.^60^

However, as discussed earlier, only one of these named officials – R.S. Hoon, head of the Indian Armed Forces Medical Services – actually served in Bhutan. *Smallpox and its Eradication*, quite misleadingly, refers to J. Kostrzewski of the Polish Academy of Sciences as the Chair for the commission for India and Bhutan; while he did play this role in India, he had no connection with certification activities inside the kingdom. To be fair to the authors of *Smallpox and its Eradication*, a marginally more transparent description of events was provided some pages later; however, even this analysis, in the form of the paragraph quoted below, did not provide a critical analysis of how work was actually carried out in Bhutan:

An International Commission consisting of 16 members from 16 countries visited Bhutan, India and Nepal in March–April 1977; groups of Commission members visited Bhutan from 28 to 30 March, Nepal from 6 to 13 April and India from 6 to 20 April. For political reasons, Bhutan and India were certified separately. Since at that time only persons of Indian nationality were allowed to visit Bhutan, Lieutenant General R.S. Hoon, an epidemiologist serving in the Indian Defence Forces and a member of the International Commission, visited Bhutan together with Dr [R.N.] Basu, after which he participated in the investigation of the adjacent Indian state of Arunachal Pradesh.^61^

Given the riches available in the WHO’s smallpox eradication archives, the information provided in the paragraph above can be charitably described as being incomplete. If one wished to be less munificent, it is entirely possible to argue that the publication is suffused with politically imposed half-truths, reliant on a smallpox certification report that had been based on the partial quotation of the available information. Although there are stray and rather weak references to collaborations with WHO frameworks in these documents, unpublished reports and communications available in the smallpox eradication archives in Geneva leave us in little doubt that the Indian government and its armed forces monopolised the power to make administrative towards explaining why the interpretational biases visible in the report titled ‘Operation Smallpox Zero’, and the potted history of Bhutan’s smallpox eradication programme contained within it were replicated almost to the letter in subsequent assessments prepared by the ‘international’ certification commission.^63^ These developments suited two interest groups: those who controlled the levers of power in that region and those who were keen not to be seen to be upsetting the political applecart.

## Concluding Comments

*Smallpox and its Eradication* urges us to accept the argument that the WHO’s attention was only directed at Bhutan’s programme after the dramatic problems faced in India were brought under control. The evidence presented here suggests a substantially different narrative. A group of medical volunteers and WHO officials were interested in dealing with the conditions existing in the kingdom in 1969, but were prevented from carrying out their plans to start a smallpox immunisation campaign in 1970. Another group of WHO officials, who were willing to accede to Indian demands that no international workers be allowed into Bhutan, were responsible for this impasse. Although concerns were raised about the transmission of smallpox from India to Bhutan, and evidence was unearthed locally about a major outbreak in 1966, appeals from the WHO HQ for investigation into these developments were dismissed in New Delhi. Senior officials at WHO HQ and WHO SEARO were soon united in falling into line, as they did not want to antagonise the Indian authorities and endanger their commitment to the country’s smallpox eradication programme. The WHO’s subsequent absence from Bhutan was publicly justified through figures that suggested negligible levels of variola incidence in the kingdom. This epidemiological data was not always accepted by WHO officials active in the districts of eastern and north-eastern India, who kept worrying about reports of smallpox cases in Bhutan that they were unable to investigate and record. These fears – and their repeated pleas for permission to assess the situation across Himalayan South Asia – were disregarded in a situation where WHO HQ and WHO SEARO were unable to convince the Indian government to change their policy in relation to the organisation’s employees. The result was a database that presents a most benign description of smallpox epidemiology in Bhutan; the creators of this bank of information were then empowered to certify their accuracy and then use these calculations to certify that smallpox had been eradicated in the kingdom.

The lack of transparency in the entire process of certification was so acute that the authors of *Smallpox and its Eradication* seemed to feel the need to purge some part of their conscience by acknowledging the existence of some unreported outbreaks in Bhutan. However, this introspection was momentary and was quickly followed by assurances that the unrecorded cases of variola had not caused wider epidemics.^64^ The material available in the archives suggests that few WHO personnel slaving away in the districts of north-eastern India shared this retrospective confidence in 1973 and 1974. These doubts – and the viewpoints that underpinned them – do not receive a mention in adulatory narratives of the worldwide eradication of smallpox, which have generally avoided discussing the limited influence of international health agencies within nations such as India and Bhutan, and other parts of the South Asian subcontinent. Such interpretative trends raise wider historical and historiographical issues that are deserving of detailed engagement.

A striking tale of human endeavour that put an end to a disease that was widely feared, the worldwide eradication of smallpox has been made all the more remarkable in retrospective retellings by participants that have privileged the roles played by certain organisations, individuals, plans and approaches.^65^ Easily malleable to heroic accounts that end up worshipping the contributions of relatively few individuals, these works have highlighted the importance of a finite set of ideas.^66^ Promoted by a rich list of publications, and involving authors who have occupied influential positions in public policy and international agencies, these participant histories have been presented as unproblematic, accurate sources of information. This explains, perhaps, why so many academics, from a variety of disciplinary backgrounds, have been so uncritical about using the contents of these narratives.^67^ The relationship between the participant historian and the unquestioning academic, who often have much in common in relation to their simplistic understandings about the unidirectional progress of medical science and its supposedly universal beneficence, has been harmonious and deeply symbiotic. They have often produced mutually sustaining narratives that have moved further away, with every retelling, from the many complexities that existed on the ground in diverse locales.^68^ Thus, all too often, subjective viewpoints presented by a small set of participants are portrayed by supposedly dispassionate academics as value-free facts. The insights provided by the vast historical collections of day-to-day communications, reports, personnel files, minutes of meetings and multiple drafts of publicity materials go unrecorded in these exercises of blind replication.^69^

Yet, this body of writing cannot be held solely responsible for inhibiting the presentation of nuanced, culturally and politically sensitive histories of smallpox eradication. Another intellectual tradition has been equally complicit in sustaining over-simplified understandings of the programme. This genre of work is characterised by a willingness to accept a number of generalisations offered by participant histories: about the operational unity of the eradication programme, as well as the capacity of a small number of international workers to ensure that their ideas and priorities were implemented across the board.^70^ Some scholars working within this intellectual tradition have adopted this approach to present generalisations about the homogeneity of attitudes within the ranks of the CDC and their supposed ability to overwhelm alternative viewpoints inside the WHO and national governments.^71^ Others have adopted a different approach. These scholars are much more transparent about using historical data sets to come up with generalisations that strengthen their arguments in contemporary debates. This type of work proceeds, roughly speaking, in three stages. The initial step is to accept claims made in participant histories about the capacity of a handful of people to enforce their priorities in the field. This is then followed by analyses that show how their actions harmed competing projects. The final step involves highlighting the worth of the views and actions of people who supported opposing programmes, such as those of primary and universal healthcare, and their criticisms of the smallpox eradication campaign.^72^

Analytical rigour becomes a prime victim when such approaches are adopted, as data is chosen selectively to buttress pre-determined arguments and contradictory evidence is downplayed or ignored. Indeed, the shared proclivity of those locked in historiographical arguments to focus on the voices of a small set of people, usually based inside North American and European institutions, causes them to minimise the significance of alternative stances and activities inside the diverse locales where projects conceived internationally were actually implemented. It is, therefore, no accident that a vast majority of these historians identify actors from North America and Europe as being the main source of leadership in the smallpox eradication programme and other international health projects; incredibly few officials from Asia, Africa and Latin America seem to make the cut in these global leadership stakes. The flip side of this approach is equally blinkered. Workers drawn from the central governments of countries with active smallpox eradication campaigns are generally presented as people who helped implement ideas brought in from the outside. This is followed by the creation of further hierarchies of worth, which generally place personnel working in the smaller towns and rural areas at the very bottom of the scale; these individuals are portrayed as voiceless, devoid of imagination and mostly incapable until introduced to the ideas advocated by international officials associated to the WHO.

The evidence presented in this article suggests the usefulness of adopting a different, more open-minded approach, one that is less accepting of the demands of current politics, and not mired in assumptions about the superiority of the intellect of specific races and class backgrounds. The methodology used here underlines the usefulness of studying a multiplicity of voices through the systematic study of a range of unpublished materials, which can reveal unexpected administrative trends and activities that mostly go unreported in official histories and memoirs. Alternative, little-known perspectives provide us with rich insights into the complex negotiations that took place inside and between nations, and their localities, as well as the intricate ways in which they became entwined with international deliberations. In many cases, as we have seen in this study of Bhutan and its links with India, international players such as the WHO and CDC were largely excluded from consultations and there was very little that these agencies could do to change existing arrangements. In Bhutan’s case, Indian political and military interests proved the most powerful determinant in the shaping of a national smallpox eradication programme, the measurement of its successes, and the ways in which its contributions to the worldwide campaign were described. In this context, epidemiology remained a less than exact science; data collection, collation and reporting were deeply political acts, subservient to the act of developing a very specific interpretation of what had happened in Bhutan.

It would, thus, be both simplistic and deeply ahistorical to assume that all the national smallpox eradication programmes in South Asia were more or less the same. Understanding the variations and dynamisms that mark the relationships between international personnel, national workers, political actors, community organisations and the social formations all of them claimed to represent is crucial to unpicking a truly elaborate history. If history is ever to play a preparatory role in the design and implementation of contemporary policy, rather than just being simply deployed as a propaganda tool, the past needs to be studied and understood in all its wondrous intricacy. Restating this point is particularly pertinent at this time, when pleas are being made, from within diverse settings, that we remember the lessons of the smallpox eradication programme. Some of the arguments being made have sought to justify contemporary causes such as the Global Polio Eradication Initiative and have, therefore, been tempered by political expediency.^73^ Some of the more self-effacing and thoughtful participants of the smallpox eradication programme have approached the issue from a different angle. By highlighting the need to remember the value of engagements with community structures, the roles played by local workers and the need for health system strengthening, they force us to ponder what kind of history can best inform contemporary health initiatives.^74^ The idealised historical narratives that bear little resemblance to the conditions that existed on the ground in the past? Or, the studies that chronicle and consider a most intricate patchwork of international, regional and national eradication programmes, by studying variations in ideologies and activities across locales? The latter approach seems the most honest way of celebrating the contributions of the many hundreds of thousands of local workers who sustained and often advised the few thousand people who agreed to serve as international civil servants in association with WHO frameworks.^75^

## Footnotes

1. For a historical explication that moves from describing origins in Edward Jenner’s discovery of a smallpox vaccine in 1796, to advances in smallpox control within the Pan American Health Organisation region, followed by a cursory reference to the WHO and India, and then to a detailed treatment of the Western and Central African smallpox eradication programmes, see William H. Foege, J.D. Millar and D.A. Henderson, ‘Smallpox Eradication in West and Central Africa’, *Bulletin of the World Health Organization*, 52 (1975), 209–22.PMC23663581083309

2. This interpretation of historical progression appears largely undiminished in Foege’s recent memoir, although the book does acknowledge the importance of the campaigns in South Asia. See William H. Foege, *House on Fire: The Fight to Eradicate Smallpox* (Berkeley, CA: University of California Press, 2011). The CDC were, of course, also involved in East Pakistan, but this seems to be ignored or downplayed in the heroic tropes about its contributions to smallpox eradication. For a masterful treatment of the CDC’s workers in South Asia, see Paul Greenough, “‘A Wild and Wondrous ride”: CDC Field Epidemiologists in the East Pakistan Smallpox and Cholera Epidemics of 1958’, *Ciencia & Saude Coletiva*, 16, 2 (2011), 491–501.10.1590/s1413-8123201100020001221340324

3. Things are rather more complicated in relation to histories of the West African – and other regional – programmes that involved Henderson as an author. In the WHO’s official history, of which he was co-author, an analysis of the Western and Central African programme appears in the seventeenth chapter, after descriptions of what happened in South America, Indonesia, Afghanistan and Pakistan, India and ‘the Himalayan area’ and, not least, Bangladesh. See F. Fenner, D.A. Henderson, I. Arita, Z. Jezek and I.D. Ladnyi, *Smallpox and its Eradication* (Geneva: WHO, 1988). In his recent memoir, the African case study appears in the fifth chapter, after an assessment of Brazil and Indonesia and before an analysis of India and Nepal. See D.A. Henderson, *Smallpox: The Death of a Disease* (Amherst, MA: Prometheus Books, 2009).

4. An exceptionally honest appraisal of field conditions in South Asia is provided in Alan Schnur, ‘Innovation as an Integral Part of Smallpox Eradication: A Fieldworker’s Perspective’, in S. Bhattacharya and S. Messenger (eds), *The Global Eradication of Smallpox* (New Delhi: Orient BlackSwan, 2010), 106–50. For a frank analysis of the autonomy of its Regional Offices and the ways in which they were able to challenge the WHO HQ, refer to the recording of a lecture on smallpox eradication by D.A. Henderson, London, 30 May 2007, available at: http://www.york.ac.uk/history/research/majorprojects/smallpox-eradication/audio/henderson/

5. Whilst the existing work on India and Bangladesh recognises the many complexities of the conditions on the ground, they do not refer to the conditions existing in the smaller political units in the neighbourhood or the effects of the influence of one nation over another. See, for instance, Sanjoy Bhattacharya, *Expunging Variola: The Control and Eradication of Smallpox in India, 1947–77* (New Delhi: Orient Longman, 2006) and Paul Greenough, ‘Intimidation, Coercion and Resistance in the Final Stages of the South Asian Smallpox Eradication Campaign, 1973–1975’, *Social Science & Medicine*, 41, 5 (1995), 633–45.10.1016/0277-9536(95)00035-67502097

6. BasuR.N., JezekZ. and WardN.A., The Eradication of Smallpox from India (New Delhi: World Health Organization South East Asia Regional Office, 1979), 93

7. Fenner *et al*, *Smallpox and its Eradication*, 802.

8. For an effective treatment of the formation, composition and activities of the BSC, see Awadesh Coomar Sinha, *Himalayan Kingdom Bhutan: Tradition, Transition and Transformation* (New Delhi: Indus, 2001). A full transcript of the India Bhutan Friendship Treaty of 2007 is available at: http://www.satp.org/satporgtp/countries/india/document/papers/indiabhutan.htm (accessed on 2 May 2012).

9. Michael Hutt, ‘Being Nepali without Nepal: reflections on a South Asian diaspora’, in D.N. Gellner, J. Pfaff-Czarnecka and J. Whelpton (eds), *Nationalism and Ethnicity in a Hindu Kingdom: The Politics of Culture in Contemporary Nepal* (Amsterdam: Harwood Academic Publishers, 1997), 101–44. For a more charitable, uncritical but detail-rich assessment of Tibetan enclaves inside Bhutan, see ‘Tibetan Settlements – Bhutan’, Department of Home, Central Tibetan Administration, Dharamshala, India, at:http://www.centraltibetanreliefcommittee.org/settlements/bhutan/settlements-in-bhutan.html (accessed on 26 December 2012).

10. A detailed assessment of US and Indian alliances in relation to covert intelligence in Himalayan South Asia, including territories within north-eastern India (Sikkim and the North Eastern Frontier Agency/Arunachal Pradesh), Nepal and Bhutan is available in Kenneth Conboy and James Morrison, *The CIA’s Secret War in Tibet* (Lawrence: University Press of Kansas, 2002).

11. For a detailed analysis of ethnic politics in Bhutan and Nepal, see Michael Hutt, *Unbecoming Citizens: Culture, Nationhood, and the Flight of Refugees from Bhutan* (New Delhi: Oxford University Press, 2003).

12. KharatR., ‘Indo-Bhutan relations: strategic perspectives’, in K. Warikoo (ed.), Himalayan Frontiers of India: Historical, Geo-political and Strategic Perspectives (Abingdon: Routledge, 2009), See also *Bhutan: Foreign Policy and Government Guide: Volume 1: Strategic Information and Developments* (Washington DC: International Business Publications, 2011).

13. WardMichael and JacksonFrederic, ‘Medicine in Bhutan’, Lancet, 285, 7389 (1965), 811–813 Recent historical scholarship on Bhutan’s health services and disease profiles has been mainly dependent on the retrospective recollections, published or otherwise, of an extremely small sample of people who have been identified by as major figures in Bhutanese medicine and public health. These memories, which have been gleaned mainly through a series of undemanding interviews, are usually reported uncritically. For a representative example, see Alex McKay, *Their Footprints Remain: Biomedical Beginnings across the Indo-Tibetan Frontier* (Amsterdam: Amsterdam University Press, 2007).4165097

14. Piece Gardner, ‘Partnership for medical progress in Bhutan’, no date, 3, Folder 55, Box 184, World Health Organization Headquarters Archives, Geneva (WHOHA).

15. Pierce Gardner, ‘Supplement Report – BHUTAN’, *c*1969, Folder 55, Box 184, WHOHA.

16. A seasonal increase in diarrhoea was reported between the months of May and July. Gardner, ‘Supplement Report’, WHOHA.

17. *Ibid*

18. Gardner, ‘Partnership for medical progress in Bhutan’, 3, WHOHA.

19. *Ibid.*, 1–4, WHOHA. Apart from Gardner, the proposed team was to include Ms Jo Ann Silverstein (a US citizen trained in French, government studies and mathematics), Dr Samuel Silverstein (a US citizen then Assistant Professor at the Department of Cellular Immunology, Rockefeller University, New York, and Jo Ann Silverstein’s husband) and Dr Michel Bernard Vallotton (a Swiss citizen who was then Chef de Clinique Scientifique at the Clinique universitaire de Medecine et Laboratoire de Physiopathologie Clinique in Geneva). Appendix to *ibid*

20. *Ibid.*, 5–6.

21. *Ibid.*, 6–7.

22. Gardner, ‘Supplement Report’, WHOHA.

23. Letter number 2–10 (TCI)/69 to P. Gardner, Camp Thimpu – Bhutan, from Minister for Trade, Commerce and Industries, 16 June 1969, Folder 55, Box 184, WHOHA.

24. The British government and its intelligence services were monitoring the political situation in Bhutan during this period, not least as the offer of military aid from the UK to the kingdom was being considered. For an assessment of trends between 1959 and 1965, see DO 164/73, DO 196/520 and DO 196/521, National Archives of Britain, Kew, UK (NAB). India’s defeat in its war with China led to greater levels of UK and US military assistance; see PREM 11/4857, NAB. This caused wider regional tensions, often involving countries such as Pakistan, which objected in 1964 and 1965 to the provision of this aid to India and Bhutan; see DO 164/134, NAB.

25. Letter to D.A. Henderson, Chief, Smallpox Eradication Unit, WHO HQ, Geneva, from P. Gardner, Assistant Professor of Medicine, The J. Hillis Miller Health Center, University of Florida, USA, 23 April 1969, Folder 55, Box 184, WHOHA.

26. Letter to D.A. Henderson, Chief, Smallpox Eradication Unit, WHO HQ, Geneva, from P. Gardner, Assistant Professor of Medicine, The J. Hillis Miller Health Center, University of Florida, USA, 19 May 1969, Folder 55, Box 184, WHOHA.

27. Letter to P. Gardner, Assistant Professor of Medicine, The J. Hillis Miller Health Center, University of Florida, USA, from D.A. Henderson, Chief, Smallpox Eradication Unit, WHO HQ, Geneva, 29 May 1969, Folder 55, Box 184, WHOHA.

28. Letter to P. Gardner, Assistant Professor of Medicine, The J. Hillis Miller Health Center, University of Florida, USA, from D.A. Henderson, Chief, Smallpox Eradication Unit, WHO HQ, Geneva, 19 June 1969, Folder 55, Box 184, WHOHA.

29. Letter to P. Gardner, Assistant Professor of Medicine, The J. Hillis Miller Health Center, University of Florida, USA, from D.A. Henderson, Chief, Smallpox Eradication Unit, WHO HQ, Geneva, 2 July 1969, Folder 55, Box 184, WHOHA.

30. Memorandum titled ‘Vaccination – Kingdom of Bhutan’ to Regional Director, WHO SEARO, New Delhi, from D.A. Henderson, Chief, Smallpox Eradication Unit, WHO HQ, Geneva, 2 July 1969, Folder 55, Box 184, WHOHA.

31. Letter to P. Gardner, Assistant Professor of Medicine, The Children’s Hospital Medical Center, Massachusetts, USA, from D.A. Henderson, Chief, Smallpox Eradication Unit, WHO HQ, Geneva, 2 July 1969, Folder 55, Box 184, WHOHA.

32. For discussions about the struggles faced by the WHO HQ and WHO SEARO in retaining the support of Indian federal and state governments, see Letter to C. Mani, Regional Director, WHO SEARO, New Delhi from D.A. Henderson, Chief, Smallpox Eradication, WHO HQ, Geneva, 4 August 1967, File 416, Box 193, WHOHA; letter to the Surgeon General, United States Public Health Service, Bethesda from D.A. Henderson, Chief, Smallpox Eradication, WHO HQ, Geneva, 6 September 1967, File 416, Box 193, WHOHA; memorandum to Regional Director, WHO SEARO, New Delhi from D.A. Henderson, Chief, Smallpox Eradication, WHO HQ, Geneva, 5 February 1968, File 416, Box 193, WHOHA; memorandum to Regional Director, WHO SEARO, New Delhi from D.A. Henderson, Chief, Smallpox Eradication, WHO HQ, Geneva, 24 October 1968, File 416, Box 193, WHOHA; and, not least, personal letter to A. Oles, WHO SEARO, New Delhi from D.A. Henderson, Chief, Smallpox Eradication, WHO HQ, Geneva, 14 September 1970, File 416, Box 193, WHOHA.

33. Memorandum number S2/370/3 (SEARO) to Regional Director, WHO SEARO, New Delhi, from D.A. Henderson, Chief, Smallpox Eradication Unit, WHO HQ, Geneva, 20 February 1970, Folder 55, Box 184, WHOHA.

34. For memoranda and telegrams about smallpox incidence and control measures in Bhutan during the 1970s, exchanged between WHO HQ, the smallpox eradication unit in WHO SEARO and WHO SEARO’s Regional Director’s office, see File 826, Box 192, WHOHA.

35. P.W. Samdup, ‘A report on the present smallpox status in Bhutan’, from Inter-Country Seminar on Surveillance in Smallpox Eradication, New Delhi, 30 October–2 November 1972, File SE/WP/72/18, WHOHA. This report can also be accessed online from: https://extranet.who.int/iris/restricted/handle/10665/68096

36. Gardner acknowledged the damaging impact of the monarch’s death on the project in an e-mail to the author of this paper. Personal communication from Pierce Gardner, USA, to Sanjoy Bhattacharya, 15 May 2012.

37. For an incisive analysis of the war that has stood the test of time, see Onkar Marwah, ‘India’s Military Intervention in East Pakistan, 1971–1972’, *Modern Asian Studies*, 13, 4, 1979, 549–80. A resource created by George Washington University, USA, makes declassified American government papers on this subject available to researchers. Sajit Gandhi (ed.), ‘The Tilt: The US and the South Asian Crisis of 1971’, National Security Archive: Electronic Briefing Book No. 79, 2002 (accessible at: http://www.gwu.edu/~nsarchiv/NSAEBB/NSAEBB79/).

38. Conboy and Morrison, *The CIA’s Secret War in Tibet* There is other evidence indicating that the Nixon administration was working secretly to improve relations with China at this time, which caused it to support Pakistan; this would also explain a reduction of CIA involvement with the covert Tibetan militias. Gandhi, ‘The Tilt’.

39. Personal letter to D.A. Henderson, Chief, Smallpox Eradication, WHO HQ, Geneva, from L.B. Brilliant, WHO SEARO, New Delhi, 20 July 1973, File 388, Box 194, WHOHA.

40. David Myard, ‘Sadruddin Aga Khan and the 1971 East Pakistani Crisis: Refugees and Mediation in Light of the Records of the Office of the High Commissioner for Refugees’, *Global Migration Research Paper (The Graduate Institute, Geneva)*, 1 (2010) 1–65.

41. Memorandum for the record, by D.A. Henderson, Chief, Smallpox Eradication, WHO HQ, Geneva, 7 March 1973, File 830, Box 194, WHOHA.

42. Personal letter to D.A. Henderson, Chief, Smallpox Eradication Unit, Geneva, from L.B. Brilliant, WHO SEARO, New Delhi, 8 August 1973, File 388, Box 194, WHOHA.

43. Letter from N. Grasset, Smallpox Eradication Unit, WHO SEARO, New Delhi, to Jock (?), WHO HQ, Geneva, *c*November 1974, File 388, Box 194, WHOHA.

44. Letter from I. Arita, Smallpox Eradication Unit, WHO HQ, Geneva, to C. de Amaral, WHO SEARO, India, 20 December 1974, File 388, Box 194, WHOHA.

45. Memorandum, WHO SEARO, *c*1973, File 826, Box 192, WHOHA.

46. Letter to D.A. Henderson, Chief, Smallpox Eradication, WHO HQ, Geneva, from WHO SEARO, New Delhi, 12 December 1973, File 388, Box 194, WHOHA.

47. Meeting notes, WHO SEARO, *c*1972, *c*1973, *c*1974 and *c*1975, File 826, Box 192, WHOHA. See also minutes of the third progress review meeting of the states/union territories of the Eastern region, *c* 1973–1974, File 834, Box 199, WHOHA.

48. Foreword by Dr T. Tobgyel, Secretary General, Ministry of Development, Royal Government of Bhutan, Thimphu, Bhutan, 15 March 1977, in ‘Operation Smallpox Zero Bhutan’, Royal Government of Bhutan, Directorate of Health Service, *c*1977, File 55, Box 184, WHOHA. Dr J.L. Kole was an Indian Public Health Official, who was seconded to Bhutan to continue its malaria eradication programme with the help of Indian aid.

49. *Ibid.*, 5.

50. *Ibid.*, 10–11.

51. *Ibid.*, 12.

52. *Ibid.*, 12–13.

53. *Ibid.*, 13.

54. *Ibid.*, 14.

55. *Ibid.*, 20–27.

56. *Ibid.*, 25.

57. Restricted document titled ‘Eradication of Smallpox in Bhutan: Report of the International Smallpox Assessment Commission, April 1977’, File 55, Box 184, WHOHA, 1.

58. *Ibid.*, 2.

59. Fenner *et al*, *Smallpox and its Eradication*, 1126.

60. Seen as a group, the list looks impressively international. It included the following personages and institutions: Dr J. Cervenka, Chief (Epidemiology), Institute of Epidemiology and Microbiology, Bratislava, Czechoslovakia; Dr W.A.B. de Silva, Deputy Director (Planning), Ministry of Health, Colombo, Sri Lanka; Dr F. Fenner, Director, Centre for Resource and Environmental Studies, The Australian National University, Canberra, Australia; Dr H. Flamm, Institute of Hygiene, University of Vienna, Austria; Lt. -Gen. R.S. Hoon, Director-General, Armed Forces Medical Services, New Delhi, India; Dr T. Kitamura, Chief, Division of Poxviruses, National Institute of Health, Tokyo, Japan; Dr W. Koinange, Director, Division of Communicable Disease Control, Ministry of Health, Nairobi, Kenya; Dr J. Kostrzewski, Secretary, Medical Section, Polish Academy of Sciences, Warsaw, Poland; Dr H.B. Lundbeck, Director, National Bacteriological Laboratory, Stockholm, Sweden; Dr A.M. Mustaqul Huq, Director of Health Services (Preventive), Ministry of Health, Dhaka, Bangladesh; Dr D.M. Mackay, Ross Institute of Tropical Hygiene, London School of Hygiene and Tropical Medicine, London, UK; Dr M.F. Polak, Scientific Officer, Faculty of Medicine, Catholic University, Nijmegen, Netherlands; Dr R. Roashan, President, Foreign Relations Department, Ministry of Public Health, Kabul, Afghanistan; Dr D.J. Sencer, Director, Center for Disease Control, Atlanta, GA, USA; Dr U Thein Nyunt, Director, Disease Control, Ministry of Health, Rangoon, Burma; and Dr V.M. Zhdanov, Director, Institute of Virology, Academy of Medical Sciences, Moscow, USSR. *Ibid.*, 1142.

61. Fenner *et al*, *Smallpox and its eradication*, 1186–7.

63. Restricted document titled ‘Eradication of Smallpox in Bhutan: Report of the International Smallpox Assessment Commission, April 1977’, File 55, Box 184, WHOHA, 2–3.

64. Fenner *et al*, *Smallpox and its eradication*, 802.

65. Some institutions and their officials have been better organised than others about creating records of their contributions to the worldwide smallpox eradication programme. See, for instance, a website titled ‘Global Health Chronicles: Smallpox’, which was put together by the CDC and Emory University (the latter hosts a large number of retired CDC officials). This can be accessed at: http://globalhealthchronicles.org/collections/show/1/

66. An early example of a history saturated with heroic accounts of the contributions of a handful of people can be seen in Donald R. Hopkins, *The Greatest Killer: Smallpox in History* (Chicago: Chicago University Press), 1983. More measured, albeit largely celebratory, accounts are available in Foege, *House on Fire*, Henderson, *Smallpox*, and Isao Arita, *The Smallpox Eradication Saga: An Insider’s View* (New Delhi: Orient BlackSwan, 2010).

67. See, for instance, Ian Glynn and Jennifer Glynn, *The Life and Death of Smallpox* (Cambridge: Cambridge University Press, 2004) and Gareth Williams, *Angel of Death: The Story of Smallpox* (Basingstoke: Palgrave McMillan, 2010).

68. The sustenance that these historical interpretations provide each other is powerfully exemplified by the mutual, public adoration between the works of Ian and Jennifer Glynn, and Frank Fenner and Donald Henderson. Whilst the Glynns make wide-ranging, uncritical use of retrospective histories of smallpox eradication authored or co-authored by Fenner and Henderson, the latter’s promotion of *The Life and Death of Smallpox* in its dust jacket is self-explanatory.

69. The most obvious examples of this are visible in Gareth Williams’s unquestioning use of data available in the WHO’s official histories and memoirs prepared by campaign participants, especially Donald Henderson’s recent book to construct a teleological account of the achievement of smallpox eradication. Williams, *Angel of Death*

70. This academic tradition, closely allied to national and international people’S health movements, has consistently advocated the need to focus state funding on the creation general healthcare facilities. For a public enagement-oriented piece by an influential academic thinker associated to this tradition, see Debabar Banerji, ‘The Battle for Total Health Care’, *India International Centre Quarterly*, 14, 3 (1987), 119–28.

71. Harish Naraindas, ‘Charisma and Triage: Extirpating the Pox’, *Indian Economic and Social History Review*, XL, 4 (2003), 425–58.

72. There is great variation in the quality of research and analysis in this genre of scholarship. One of the best pieces of scholarship available is Anne-Emanuelle Birn, ‘Small (pox) Success’, *Ciencia & Saude Coletiva*, 16, 2 (2011), 591–7.10.1590/s1413-8123201100020002221340334

73. Powerful examples of this are available in the messages transmitted from within departments of the Bill & Melinda Gates Foundation. See, for instance, Tachi Yamada, ‘Smallpox Eradication Taught Us How to Fight Polio: Now We Need to Win the Battle’, 26 August 2010 (accessible at: http://www.impatientoptimists.org/Posts/2010/08/Smallpox-Eradication-Taught-Us-How-to-Fight-Polio-Now-We-Need-to-Win-the-Battle). For a fearless assessment of the origins of the global polio eradication programme and the use subjective historical narratives to advocate it, see William Muraskin, *Polio Eradication and its Discontents: A Historian’s Journey Through an International Public Health (Un)Civil War* (New Delhi: Orient BlackSwan, 2012).

74. See, for example, Daniel Tarantola and Stanley Foster, ‘From smallpox Eradication to Contemporary Global Health Initiatives: Enhancing Human Capacity towards a Global Public Health Goal’, *Vaccine*, Supplement 4, 29 (2011), 135–40.10.1016/j.vaccine.2011.07.02722185838

75. An approach that is best served by rigorous research in the archives, it helps prepare a methodology that does not rely on the selective reading of relatively small sets of published materials. This allows us to avoid a mindless dependence of views expressed inside institutions in Europe and North America, a tendency that can be seen in a recent working paper produced by Eleanor Davey, in association with John Borton and Matthew Foley. E. Davey, *A History of the Humanitarian System: Western Origins and Foundations* (London: Humanitarian Policy Group, 2013). This publication is accessible at: http://www.odi.org.uk/sites/odi.org.uk/files/odi-assets/publications-opinion-files/8439.pdf).

